# Risk for Coccidioidomycosis among Hispanic Farm Workers, California, USA, 2018

**DOI:** 10.3201/eid2607.200024

**Published:** 2020-07

**Authors:** Stephen A. McCurdy, Catherine Portillo-Silva, Carol L. Sipan, Heejung Bang, Kirt W. Emery

**Affiliations:** University of California, Davis, California, USA (S.A. McCurdy, H. Bang);; University of California, Merced, California, USA (C. Portillo-Silva, C.L. Sipan);; Kern County Public Health Services Department, Bakersfield, California, USA (K.W. Emery)

**Keywords:** coccidioidomycosis, farm workers, Hispanics, occupational health, California, United States, fungi, Coccidioides

## Abstract

To determine occupational risk factors for coccidioidomycosis among adult Hispanic outdoor agricultural workers in California, USA, we conducted a case–control study of workers seen at the Kern County medical facility and referred to the public health laboratory for coccidioidomycosis serologic testing. Participants completed an interviewer-administered health and work questionnaire. Among 203 participants (110 case-patients with positive and 93 controls with negative serologic results), approximately half were women, and more than three quarters were born in Mexico. Associated with coccidioidomycosis were self-reported dust exposure and work with root and bulb vegetable crops. A protective factor was leaf removal, an activity associated with grape cultivation. We conclude that subjective dust exposure and work with root and bulb vegetable crops are associated with increased risk for coccidioidomycosis among Hispanic farm workers. The agricultural industry should evaluate and promote dust-reduction measures, including wetting soil and freshly harvested products.

Coccidioidomycosis (Valley fever or San Joaquin Valley fever) is a pulmonary and systemic infection that results from respiratory exposure to aerosolized arthroconidia spores of soil-dwelling species of *Coccidioides* fungi (*1*). *Coccidioides* fungi and coccidioidomycosis are strongly associated with the semiarid climate of the Lower Sonoran life zone of the southwestern United States and parts of Mexico, Central America, and South America. Most US cases occur in Arizona and California. However, the fungus and locally acquired cases have recently been reported as far north as Washington state, potentially related to changes in climate and land use patterns (*2*,*3*). In California, the species most often implicated in human infection is *C. immitis*, whereas in Arizona it is *C. posadasii* (*2*,*3*).

After an incubation period of ≈1–3 weeks, most infected persons experience few or mild symptoms, and the condition usually resolves within weeks or months, often unrecognized (*4*,*5*). Approximately 40% of infected persons experience an influenza-like illness with cough, fever, and fatigue that typically resolves without treatment. Approximately 1% of cases involve dissemination to skin, bone, meninges, and other tissues (*6*); patients with disseminated disease require long-term antifungal therapy and may die (*7*). Risk factors for disseminated disease include male sex, age >60 years, pregnancy, immunocompromise, and African and Filipino ancestry (*8*).

Case identification is based on clinically compatible illness with confirmatory laboratory evidence or skin-test conversion (*9*). Local health departments may find it impractical to obtain clinical information and thus may identify cases solely on the basis of laboratory results (*4*). Because most cases are subclinical, public health surveillance substantially underestimates infection risk. McCotter et al. estimated that the true number of cases is ≈4–6-fold greater than that captured by public health surveillance (*2*).

Residence in or visits to coccidioidomycosis-endemic areas may lead to exposure and infection. The largest outbreaks have been associated with natural phenomena. A December 1977 dust storm in California’s San Joaquin Valley (an area of high coccidioidomycosis endemicity, from which the disease derives its common name) resulted in a ≥10-fold increase in incidence in 15 of the state’s 58 counties (*1*,*10*). The 1994 Northridge, California, earthquake was responsible for 203 outbreak-associated cases, including 3 deaths (*11*). Occupational risk has been associated with soil-disruptive activity involving archeologists (*12*), film crews (*13*), solar power farm construction workers (*14*,*15*), roadway and construction workers (*16*,*17*), and agricultural workers (*18*). Prison inmates in the San Joaquin Valley are also at increased risk, leading to a policy of excluding inmates with nonreactive spherulin-based coccidioidomycosis skin tests from these facilities (*19*).

In recent decades, coccidioidomycosis in California has increased markedly. Since individual case reporting began in 1995, the 2 highest years on record have been 2017 (7,658 cases) and 2018 (7,515 cases) (*4*). During 2000–2013, an average of 78 deaths/year were attributed to coccidioidomycosis in California (*20*), and during 2000–2011, ≈25,000 hospitalizations were reported (*21*). The historically highest incidence among California counties has been in Kern County (2,937 cases in 2018, 323.2 cases/100,000 persons/year), and several factors may contribute. First, the county is situated within the highly coccidioidomycosis-endemic San Joaquin Valley. Second, Kern is the most productive agricultural county in the nation (*22*), so soil disruption and exposure to agricultural dust are common. Third, Kern County leads the state by having had ≈116,000 hired farm workers in 2014 (*23*). Although Hispanic origin has not been shown to be an independent risk factor for coccidioidomycosis (*8*), and to our knowledge no outbreaks in this group have been reported, ≈95% of hired crop workers in California are Hispanic (*24*) and thus represent a large occupational risk group.

Despite longstanding recognition of agricultural work as an occupational risk factor for coccidioidomycosis, little research to identify specific high-risk agricultural exposures, such as crops or tasks, has been conducted. We therefore conducted a case–control study of coccidioidomycosis in Hispanic farm workers in Kern County, California, focused on identifying occupational risks. We tested the hypothesis that subjective dust exposure is related to risk for infection and conducted exploratory analyses to identify associated crops and tasks.

## Materials and Methods

### Study Design

For this case-control study, we used data collected from June 1, 2016, through August 31, 2018. During this period, the Kern County Public Health Services Department provided a list, approximately monthly, of persons who had undergone serologic testing for coccidioidomycosis at the Kern County Public Health Laboratory after referral from Kern Medical, the county public healthcare organization where local Hispanic agricultural workers are likely to seek care. Kern Medical comprises a 222-bed teaching hospital and clinics providing primary and specialty care for an ethnically diverse population. Kern Medical has longstanding expertise with coccidioidomycosis and hosts the Valley Fever Institute (*25*) to promote education, treatment, and research on coccidioidomycosis.

### Study Sample

To assess candidacy for our study, we made up to 10 attempts to contact by telephone all persons referred from Kern Medical for coccidioidomycosis serologic testing who were >18 years of age and had >1 positive serologic test result (potential cases) and a random sample of persons with all negative serologic test results (potential controls). We invited to complete a full in-person interview those screened persons with self-declared Hispanic or Latino origin who had worked in outdoor agriculture in Kern County for >1 month in the preceding year, were not incarcerated, and were able to provide informed consent in Spanish or English. We initially excluded women who had been pregnant in the past year because Kern Medical uses coccidioidomycosis testing for screening of pregnant women rather than for diagnosis of suspected illness. However, because of low enrollment, we removed the pregnancy exclusion and considered as study candidates all persons who had undergone coccidioidomycosis serologic testing after referral from Kern Medical. Participants received a gift certificate for $15 in appreciation for their time. The University of California Davis Institutional Review Board approved and monitored the study (IRBNet ID 747508).

### Definitions

Serologic evaluation comprised 3 tests: immunodiffusion for IgM, immunodiffusion for IgG, and complement fixation (considered positive for dilutions >1:2). Case-patients were defined as persons with >1 positive serologic test result. Control status was assigned to persons with negative results for all 3 serologic tests.

### Questionnaire Development and Administration

We reviewed available survey instruments from previous outbreak investigations in Kern and San Joaquin Counties (*26*) and incorporated relevant material into our questionnaire. Colleagues in the California Department of Public Health and the Centers for Disease Control and Prevention reviewed questionnaire drafts. We pilot tested the questionnaire with a sample of 20 Hispanic agricultural workers in Kern County and excluded those persons from this report. The final questionnaire addressed demographic characteristics; health history; and agricultural work history with job start and end dates for the preceding year, weekly hours, frequency of outdoor work, crop or commodity, tasks, subjective dust exposure, and frequency of use in dusty conditions for personal respiratory protective measures and soil wetting. We separated jobs by change in location and task. After obtaining informed consent, trained study staff fluent in Spanish and English and blinded to participant case status administered the questionnaire in each participant’s home or in the study office.

### Exposure Assessment

Participants whose jobs involved a specific crop or task were considered to have been exposed to that crop or task; we added the total weeks of exposure over all jobs in the preceding year. We combined crops into 3 categories based on likely exposure to potentially infectious soil dusts: root and bulb vegetables growing underground (beets, carrots, garlic, onions, radishes, sweet potatoes), near-ground crops (blueberries, chili peppers, cotton, grapes, kale, lettuce, spinach, strawberries, tomatoes, watermelons), and tree crops (almonds, apples, apricots, cherries, kiwis, oranges and Mandarin oranges, pomegranates, pistachios). We also examined individual crops and tasks reported by >9% of participants. Dust exposure was based on response to the question: “How often did/does your work at this job generate a lot of dust?” Responses were “never/sometimes/half of the time/most of the time/always.” For final analyses, we dichotomized these responses as “never/sometimes” versus “half of the time/most of the time/always.” These responses were also used for mask use (“When working in dusty conditions, how often did/do you wear a mask?”) and frequency of soil wetting (“When working in dusty conditions, how often is/was the soil kept wet to reduce dust?”).

### Data Management and Analysis

Data from the paper questionnaires were double-entered into a computer database; discrepancies were checked against the paper questionnaire and corrected. Subsequently, a 20% sample of randomly selected questionnaires was checked against the digital database; 6 entry errors among 223 variables per questionnaire were identified and corrected (error rate <0.15%). 

We analyzed data by using Stata 15.1 (https://www.stata.com). We summarized distributions of continuous variables with either means and SDs (for approximately bell-shaped distributions) or medians and interquartile ranges (IQRs). We summarized categorical variables as percentages within each category. For group comparisons, we used the Fisher exact test for nonordinal categorical variables and the Kruskal-Wallis test for continuous and ordinal categorical variables. 

We initially examined 2-dimensional tables of case status by selected occupational and demographic characteristics and used the Stata logistic command to derive unadjusted odds ratios (ORs), with subsequent adjustments for age and sex. We assessed robustness by comparing adjusted odds ratios from the entire sample with those derived among men and women separately, after removing from analysis women reporting a pregnancy in the preceding year and persons reporting no symptoms.

## Results

### Study Sample and Demographic Characteristics:

We screened 1,803 (51%) of 3,509 persons selected from the Kern County Public Health Services Department periodic lists of coccidioidomycosis testing referrals. Reasons for not screening were wrong numbers or failure to connect after 10 attempts (≈75%), language or communication difficulty (≈15%), or the person declining to be screened (≈10%). Of those screened, 380 (21%) persons were eligible; of those, 215 (57%) completed an interview. Participation was higher for case-patients (70%) than for controls (48%; p = 0.0001). Of those interviewed, 203 (110 case-patients and 93 controls) were subsequently confirmed as eligible and included in our sample (Table 1). The major reasons for postinterview disqualification were lack of qualifying agricultural work and indeterminate serologic test results. Median time from testing to interview was 39 (IQR 25–55) days. 

**Table 1:** Selected demographic and health characteristics of 203 Hispanic farm workers evaluated for coccidioidomycosis, Kern County, California, USA, 2016–2018*

Characteristic	Case-patients, n = 110	Controls, n = 93
Sex, no. (%)		
F	46 (42)	53 (57)
M	64 (58)	40 (43)
Age, y		
Median		
M	38.7	42.0
F	38.1	32.7
IQR		
M	29.6–47.8	29.0–50.5
F	28.5–46.3	27.0–39.5
Country of birth, no. (%)		
Mexico	83 (76)	74 (80)
United States	16 (15)	16 (17)
Central America	11 (10)	3 (3)
Language used during interview, no. (%)		
Spanish	94 (85)	80 (86)
English	16 (15)	13 (14)
Years lived in United States		
Median		
M	18.7	23.2
F	18.0	16.6
IQR		
M	13.2–27.4	14.8–32.7
F	12.5–23.3	9.6–20.3
Years lived in Kern County		
Median		
M	11.2	16.8
F	14.0	11.6
IQR		
M	5.6–21.5	10.7–26.2
F	7.2–14.9	4.7–17.0
Years of education		
Median	7.5	9.0
IQR	6–12	6–12
Annual family income, US $, no. (%)		
<9,000	17 (15)	11 (12)
9,001–12,000	15 (14)	10 (11)
12,001–15,000	13 (12)	17 (18)
15,001–20,000	12 (11)	15 (16)
>20,001	24 (22)	20 (22)
Not stated	29 (26)	20 (22)
Smoker status, no. (%)		
Never	80 (73)	65 (70)
Former	27 (25)	22 (24)
Current	3 (3)	6 (6)
Selected health conditions, no. (%)		
Diabetes	20 (18)	17 (18)
Asthma	5 (5)	7 (8)
Other lung conditions	31 (28)	17 (18)
Cancer	5 (5)	7 (8)
Pregnancy†	12 (27)	39 (74)

*IQR, interquartile range. †Percentage calculations based on women only.

Women represented a greater proportion of controls (57%) than of case-patients (42%); p = 0.04). Female controls were also significantly younger than male controls and case-patients of each sex (p = 0.03). More than three quarters of participants were born in Mexico, and ≈85% completed the interview in Spanish. Median time living in Kern County was >10 years. Median annual family income was in the $15,001–$20,000 category, and most participants had completed <9 years of formal education. Pregnancy within the past year was less frequent among female case-patients (27%) than female controls (74%; p = 0.0001).

### Occupational Characteristics

Case-patients and controls were comparable for number of jobs (median 3), weeks worked in outdoor agriculture in the preceding year (median 30), and weekly work hours in the most recent job (median 48). A significantly higher percentage of case-patients (21%) than controls (9%) worked with root and bulb vegetable crops (p = 0.02; Table 2). The most frequently worked crop was grapes, worked less frequently by case-patients (58%) than controls (72%; p = 0.06). Case-patients and controls were comparable with respect to agricultural tasks, with the exception of leaf removal, which was performed significantly less frequently by case-patients (23%) than controls (40%; p = 0.01).

**Table 2:** Selected occupational exposures and associations with coccidioidomycosis among 203 Hispanic farm workers, Kern County, California, USA, 2016–2018

Characteristic	Cases, no. (%), n = 110	Controls, no. (%), n = 93	Adjusted odds ratio (95% CI)*
Self-reported dust exposure for most recent job			
Never or sometimes	32 (29)	41 (44)	Reference

Half time or more	78 (71)	52 (56)	1.9 (1.0–3.5)
Work in crop category in past year†‡			
Root and bulb vegetable crops†‡	23 (21)	8 (9)	3.0 (1.2–7.1)
Near-ground crops†§	76 (69)	76 (81)	0.5 (0.3– 1.0)
Tree crops†¶	60 (55)	41 (44)	1.4 (0.8–2.5)
Work with specified crop in past year†#			
Grapes	64 (58)	67 (72)	0.6 (0.3–1.0)
Almonds	24 (22)	17 (18)	1.1 (0.5– 2.3)
Mandarin oranges	23 (21)	19 (20)	1.1 (0.5– 2.1)
Carrots	14 (13)	5 (5)	2.9 (1.0. 8.6)
Tasks performed in past year†**			
Harvest	82 (75)	68 (73)	1.3 (0.7– 2.5)
Pruning	40 (36)	39 (42)	0.7 (0.4– 1.3)
Packing (outdoor)	39 (35)	33 (35)	1.2 (0.6– 2.1)
Leaf removal	25 (23)	37 (40)	0.4 (0.2– 0.8)
Weeding	14 (13)	10 (11)	1.3 (0.5– 3.1)
Irrigation	15 (14)	7 (8)	1.6 (0.6– 4.2)
Mask use for most recent job			
Never or sometimes	58 (53)	40 (43)	Reference
Half the time or more	51 (47)	53 (57)	0.9 (0.5– 1.7)
Soil wetting in dusty conditions for most recent job			
Never or sometimes	63 (58)	48 (52)	Reference
Half the time or more	46 (42)	45 (48)	0.8 (0.4– 1.3)

*Adjusted for age and sex. †Categories are not mutually exclusive; reference group is all participants not working in specified category. ‡Root and bulb vegetables growing underground: beets, carrots, garlic, onions, radishes, sweet potatoes. §Near-ground crops: cotton and fruits and vegetables growing above ground and near the surface, including blueberries, chili peppers, grapes, kale, lettuce, spinach, strawberries, tomatoes, watermelon. ¶Tree crops: almonds, apples, apricots, cherries, kiwi, oranges and Mandarin oranges, pomegranates, pistachios. #Limited to crops reported by >9% of participants; crops are not mutually exclusive. **Limited to tasks reported by >9% of participants; tasks are not mutually exclusive.

Always working outdoors was reported by 94% of participants. With respect to the most recent job, significantly more case-patients (71%) than controls (56%) reported dust exposure for half of the time or more (p = 0.02), whereas mask use and soil wetting in dusty conditions on a half-time or more basis were comparable among case-patients and controls, reported by approximately half of participants. Neither mask use nor soil wetting were independently associated with self-reported dust exposure. Among 117 persons reporting any mask use, 102 (87%) reported wearing a bandana, 7 (6%) using an N95 respirator, and 1 (1%) wearing a half-face mask. Women were markedly more likely than men to report mask use (77% vs. 27%; p = 0.0001).

### Associations between Occupational Exposures and Coccidioidomycosis

Age- and sex-adjusted ORs for having coccidioidomycosis (Table 2) were significantly elevated for self-reported dust exposure (OR 1.9, 95% CI 1.0–3.5) and work with root and bulb vegetables (OR 3.0, 95% CI 1.2–7.1). Exploratory analysis of hours worked with root and bulb vegetable crops did not show a dose response, although 95% CIs were wide. For work with root and bulb vegetables, the OR for work with carrots was also elevated (OR 2.9, 95% CI 1.0–8.6). ORs were reduced for those who worked with grapes (0.6, 95% CI 0.3–1.0) and leaf removal (OR 0.4, 95% CI 0.2–0.8). Mask use and soil wetting in dusty conditions were associated with modest and statistically nonsignificant reductions in ORs. Patterns were similar for men and women separately and after excluding women pregnant in the prior year (12 cases, 39 controls) and persons reporting no clinical features of coccidioidomycosis (4 cases, 16 controls).

### Clinical Characteristics

Case-patients were significantly more likely than controls to report clinical features associated with coccidioidomycosis (Table 3); adjusted ORs ranged from 2.6 (weight loss) to 4.3 (shortness of breath). The most frequent signs/symptoms among all participants were fatigue and cough. Case-patients reported significantly greater median weight loss (10 vs. 0 lb; p = 0.003) and lost workdays (18.5 vs. 0 d; p = 0.0001) than did controls. Case-patients had >2-fold increased odds of being hospitalized.

**Table 3:** Reported clinical characteristics among 203 Hispanic farm workers evaluated for coccidioidomycosis, Kern County, California, USA, 2016–2018

Clinical characteristic	Cases, no. (%), n = 110	Controls, no. (%), n = 93	Adjusted odds ratio (95% CI)*
>1 clinical characteristic	100 (91)	72 (77)	4.4 (1.4–14.2)
Fatigue or weakness	91 (83)	52 (56)	3.7 (1.9–7.2)
Cough	74 (67)	38 (41)	3.0 (1.6–5.3)
Night sweats	73 (66)	34 (37)	3.2 (1.8– 5.8)
Weight loss†	73 (66)	40 (43)	2.6 (1.4–4.7)
Fever	72 (65)	29 (31)	4.0 (2.2–7.4)
Chest pain	71 (65)	29 (31)	3.8 (2.1– 7.0)
Shortness of breath	66 (60)	25 (27)	4.3 (2.3–7.9)
Difficulty breathing	66 (60)	34 (37)	2.8 (1.5–4.9)
Hospitalized‡	52 (47)	24 (26)	2.3 (1.2–4.4)
Missed work§	88 (80)	45 (48)	4.0 (2.1–7.7)

*Adjusted for age and sex. †Median (interquartile [IQR]) weight loss, in pounds, by case-patients vs. controls 10 (0–18) vs. 0 (0–10); p = 0.003, Kruskal-Wallis test. ‡Median (IQR) nights hospitalized for case-patients vs. controls 0 (0–5) vs. 0 (0–1); p = 0.002, Kruskal-Wallis test. §Median (IQR) days of work lost by case-patients vs. controls 18.5 (2–48) vs. 0 (0–24); p = 0.0001, Kruskal-Wallis test.

## Discussion

We report the results of a case–control study that examined potential occupational risk factors for coccidioidomycosis among Hispanic agricultural workers in Kern County, California. Self-reported dust exposure was significantly associated with a near doubling of the odds of coccidioidomycosis, and odds were increased a significant 3-fold for those who worked with root and bulb vegetable crops. Leaf removal, a task almost uniquely limited to grape cultivation, was associated with a significant 60% reduction in odds. Mask use (chiefly cloth bandanas) and soil wetting was associated with modest and statistically nonsignificant reductions of odds of coccidioidomycosis. The clinical findings illustrate a heavy health burden. The most prevalent symptom was fatigue or weakness, reported by ≈80% of cases and associated with a median 18.5 days of lost work time. Those lost days accounted for ≈10% of the 213 average annual workdays for California farm workers in 2015–2016 (*24*) and represent an income loss few can afford.

The association with self-reported dust exposure is consistent with the mechanism of exposure, whereby aerosolized *Coccidioides* spores are inspired and establish infection in the lung. Thus, time spent outdoors, where wind may carry dust and spores from on site or afar, is an environmental risk for the general population in coccidioidomycosis-endemic areas and especially for persons whose work is outdoors or involves soil disruption (*27*). The largest occupational risk group is agricultural workers. In 2014, there were an estimated 829,300 hired agricultural workers in California (*23*); ≈95% were Hispanic (*24*). Thus, Kern County and the San Joaquin Valley represent the confluence of multiple factors contributing to the public health effects of coccidioidomycosis: environmental conditions favorable to growth and aerosolization of *Coccidioides* spores, predominance of agriculture with concomitant soil disruption in an outdoor work setting, and a large population of occupationally exposed persons.

With this study, we aimed to identify specific agricultural exposures and practices affecting risk. We observed a 3-fold increase in the odds of coccidioidomycosis among root and bulb vegetable crop workers, although a dose-response phenomenon was not evident. The mechanism behind the observed increased risk may relate to dust exposure during cultivation and harvest. Harvest typically is mechanized, but workers may be involved in handling and shaking off surface and subsurface dirt, potentially bearing infectious *Coccidioides* spores, from freshly harvested product as it is prepared for transport and market. Similarly, reduced risk associated with leaf removal and work with grapes may be mediated through less frequent work with other crops and tasks associated with exposure to dust from surface and subsurface dirt.

Mask use and soil wetting showed modest and statistically nonsignificant protective effects. Mask use, chiefly with a pañuelo, a cloth bandana covering the mouth and nose, is primarily reported by women, here and in other studies (*28*). Cloth bandanas are of limited effectiveness for respiratory protection (*29*), and educational campaigns should focus on limiting work under dusty conditions and promoting use of National Institute for Occupational Safety and Health (NIOSH)–approved respirators with particulate filters rated N95, N99, N100, P100, or HEPA when such work is necessary. Unfortunately, compliance may be reduced by the practical challenges of using personal respiratory protection, including discomfort and interference with speed and workflow.

A limitation of this study is low power due to sample size, limiting exploration of confounding as a possible contributor to observed associations. For example, associations between crop or task and disease risk could be confounded by sociodemographic and other factors that may channel susceptible persons to certain crops and work activities. Sample size also affected precision and subgroup analyses, yet we nevertheless found statistically significant associations for subjective dust exposure, work with root and bulb vegetables, and leaf removal and nonsignificant findings in the expected direction of effect for mask use and soil wetting. These findings suggest the need for focused studies looking at dust exposure and disease risk in association with specific crops and activities and efficacy of protective programs.

We originally planned to limit participation to persons referred for *Coccidioides* serologic testing because of clinical indications of disease. However, it was not feasible for the Kern County Public Health Services Department to document clinical features as recommended by the Council of State and Territorial Epidemiologists (*9*), and case identification was based solely on serologic test results. Moreover, women seen for obstetric care at Kern Medical are referred for coccidioidomycosis serologic testing as a screening measure. However, exploratory and sensitivity analyses, in which we examined men and women separately and removed women reporting pregnancy in the preceding year and persons without reported clinical features, did not materially change our findings.

Selection bias may also have affected our results in several ways. First, the sample was limited to persons working in Kern County. Although the study population was demographically similar to published descriptions (*24*) of California Hispanic farm workers, it is possible that our population differed from those in other locales in ways that may affect risk for coccidioidomycosis. Second, the study could not include case-patients who were asymptomatic or did not seek evaluation. Our study sample probably represents persons with more severe cases because all sought medical care from Kern Medical, the county healthcare facility. Moreover, nearly half of the case-patients reported overnight hospitalization. Thus, the epidemiologic patterns observed here may not apply to persons with milder cases that are likely to remain unrecognized. In addition, the Berkson bias (*30*) may yield observed ORs for agricultural exposures that underestimate true associations (i.e., if agricultural exposures cause pulmonary conditions other than coccidioidomycosis, such that those exposures are more prevalent in controls than they would otherwise be). Third, a low participation rate, especially among controls, may also have introduced selection bias. For example, if healthier control candidates were less likely to participate and had lower exposure levels contributing to their good health, our control sample would be biased toward higher exposures, leading to underestimation of true ORs. Alternatively, if healthier control candidates were more likely to participate and had lower exposure levels contributing to their good health, our control sample would be biased toward lower exposures, leading to overestimation of true ORs.

We were also unable to validate self-reported exposure information and clinical features. Hence, reporting bias may have affected our results, particularly if case-participants suspected that certain exposures might be important and overreported those. However, we are unaware of beliefs regarding coccidioidomycosis in this population that might affect reporting behavior, and we consider reporting bias an unlikely explanation of the observed associations.

On the basis of our findings, we recommend basic research on exposure, to include local soil sampling for *Coccidioides* spp. and area and personal respirable air sampling during harvest and other phases of cultivation, especially for root and bulb vegetable crops where exposure to surface and subsurface dust is likely. Although daunting from a practical standpoint, cohort studies of susceptible persons (i.e., based on nonreactive initial spherulin skin test) would help establish incidence and epidemiologic patterns while including persons contracting subclinical infections. Although the effectiveness of preventive recommendations remains uncertain (*8*), current recommendations (*27*,*31*) for reducing dust exposure, including wetting soil and freshly harvested products, should be followed, especially for persons working with root and bulb vegetable crops. Personal respiratory protection, using NIOSH–approved equipment, should be encouraged. Because masks are more commonly used by women, educational programs should focus on improving acceptance among men and include evaluation of program effectiveness. Programs must also account for factors interfering with education and care that are common among the Hispanic agricultural worker population, including social marginalization associated with language, culture, and legal status and low levels of health insurance (*32*).
